# The Lived Experience of Patients Utilizing Second-Generation Direct-Acting Antiviral for Treatment of Chronic Hepatitis C Virus Infection: A Phenomenological Analysis

**DOI:** 10.3390/ijerph191912540

**Published:** 2022-10-01

**Authors:** Yone de Almeida Nascimento, Luciana Diniz Silva, Djenane Ramalho de Oliveira

**Affiliations:** 1 College of Pharmacy, Center for Pharmaceutical Care Studies, Universidade Federal de Minas Gerais, Belo Horizonte 31270-901, MG, Brazil; 2 College of Medicine, Universidade Federal de Minas Gerais, Belo Horizonte 31270-901, MG, Brazil

**Keywords:** hepatitis C, chronic, qualitative research, phenomenology, Merleau-Ponty phenomenology, experience with medications

## Abstract

Hepatitis C is a global public health problem, and the aim of this study was to understand the experiences of patients with hepatitis C using second-generation antivirals. In-depth interviews were conducted with ten outpatients, cognitively capable of reporting their experience, followed up at a university clinic. Field diaries kept during the interviews were also used. The researchers carried out a thematic analysis to identify the ways in which individuals experienced their medication; then, these ways were reorganized to encompass the essential structures of the experience. The patients experienced the use of DAAs as providing resolution and it was permeated by: the experience of time—stagnant time, waiting for medication and the cure; the experience of spaces, understood as necessary and imposed spaces; the experience of relationships with others, personified by the support provided by healthcare professionals; the experience of sexuality, when patients developed several coping strategies to deal with the challenges imposed by the treatment. To conclude, increasing the knowledge about the patients’ experiences can contribute to improve the healthcare model for hepatitis C, since several patients have severe hepatic impairment, and the eradication of the virus is only one of the stages of patients’ treatments.

## 1. Introduction

Hepatitis C is considered a major public health problem in Brazil and worldwide [1,2]. In general, its evolution is slow and progressive: about 80% of individuals acquire an acute infection become chronic carriers, and about 20% of infections evolve to cirrhosis in approximately 10 to 20 years after infection [3,4]. It is currently the leading cause of liver transplantation in major world centers [5]. An estimated 71 million people are chronic carriers of the hepatitis C virus (HCV), and more than 400,000 die every year as a result of liver disease associated with the virus [6].

The treatment of chronic hepatitis C has undergone a real revolution. The standard therapy used in the last 15 years was based on a combination of pegylated interferon (PegIFN) alpha-2A or alpha-2B and ribavirin (RBV) [7]. The goal of the treatment is to achieve a sustained virological response (SVR), defined as the absence of detectable serum HCV six months after the end of treatment. SVR rates with these combinations reached 40% to 50% for patients infected with genotype 1, and 80% or more for those infected with genotypes 2 or 3 [8].

In 2011, first-generation direct-acting antivirals (DAAs) were introduced: boceprevir and telaprevir [9]. These drugs were used in combination with PegIFN and RBV and were associated with higher treatment complexity and high frequency of adverse effects [10] Consequently, three years after its approval, the therapy was no longer recommended in many countries [11].

Since 2013, therapy without the use of interferon-alpha (IFN-α) has become a reality. In 2015, the U.S. Food and Drug Administration (FDA) and the European Medical Agency (EMA) approved new DAAs: sofosbuvir; simeprevir; daclatasvir; a combination of sofosbuvir + ledipasvir; and a combination of paritaprevir + ombitasvir + dasabuvir. SVR rates reached percentages between 92% and 100% [12] and, for the first time, 90% of patients affected by chronic HCV could be cured after only three months of therapy [2,4]. However, advancement in the treatment of chronic hepatitis C also poses a challenge for many health systems around the world. Although extremely safe and effective, these new therapies are expensive [13]. In Brazil, the Ministry of Health provides the following combinations of DAAs to treat chronic hepatitis C: ledipasvir/sofosbuvir, glecaprevir/pibrentasvir and velpatasvir/sofosbuvir [14,15].

Studies aimed at describing the experience of patients with chronic hepatitis C are scarce, particularly investigations that intend to assess the experience of these patients with the use of medications. Prior studies have described the experience of patients with chronic hepatitis C during the use of antiviral therapy with the medications PegIFN and RBV [16,17,18,19,20], with the use of PegIFN + RBV + DAAs (boceprevir or telaprevir) [21,22,23]; and with new second-generation DAAs [24,25,26]. The subjective experience of using medications is individual; however, it can potentially influence health outcomes [27]. Understanding the subjective, complex and multidimensional nature of this phenomenon can result in important health implications, such as improving patients’ quality of care. However, being aware of the difficulties experienced by patients is not enough to guide reflective practice [28]. It is also important to operationalize knowledge related to the experience of using medications and this application in clinical care should not be overlooked [29,30].

Merleau-Ponty’s philosophy is based on the body, and as such, it can expand our understanding of the phenomena linked to the use of medications, considering that these products can generate deep changes in the physical and “phenomenal body” [31]. Merleau-Ponty discussed how we, as embodied subjects, experience the world and its essential structures—time, space, relationships with others and sexuality—through our experienced or phenomenal body [28,32]. Based on that, the aim of this study was to understand the experiences of patients with chronic hepatitis C using second-generation antivirals and to describe how the essential structures operate in these patients’ daily use of these medications.

## 2. Materials and Methods

### 2.1. Participants

This study was conducted in the Viral Hepatitis Outpatient Clinic, which is an outpatient care ambulatory of a metropolitan tertiary teaching hospital that admits patients for the treatment of chronic viral hepatitis. All participants were informed of the objectives of this study, and those who agreed to participate signed the informed consent form. The study was designed and conducted in accordance with the Declaration of Helsinki and was approved by the Ethics Committee of Federal University of Minas Gerais/UFMG protocol code 664,354.

To ensure the anonymity of the participants, fictitious names were used and the names of any diseases/clinical situations and the names of any medications that could identify them were removed from the transcripts.

Patients chronically infected with HCV were assessed just before starting DAA therapy (sofosbuvir, daclastavir and simpeprevir) or during antiviral treatment. Patients who were cognitively capable of reporting their experience were invited to participate. Patients with hepatic encephalopathy and/or clinical conditions associated with mental confusion were not included. The characteristics of the patients with CHC included in the study were [n = 10; mean age 52.6 ± 11.4 years; 7/10 (70.0%) females]. The mean of total household income/month was between one to three minimum wages. The Brazilian national minimum wage was BRL 880.00 ($223.45) in 2016. The participants presented a wide array of biomedical characteristics, type/number of clinical comorbidities, time of diagnosis of chronic hepatitis C, presence of complications of hepatopathy and number of previous HCV treatments.

### 2.2. Data Collection

In-depth interviews were conducted with ten individuals, between March and September 2016. After eight interviews, no new units of significance emerged [33,34,35]. The interviews were carried out individually, lasting an average of 50 min, and were recorded and then transcribed. The purpose of the interview was to allow the interviewees to describe and reveal the dimensions of their experience with DAAs. The topic guide contained the following open-ended questions: (1) “I’d like to go back to the moment you found out you had hepatitis. Tell me how your journey has been since then”; (2) “What is it like for you to use these drugs for the treatment of hepatitis? (3) “What happened during the treatment?” Based on these questions, the patients constructed a history of their disease and all the treatments they had undergone.

All the subjective aspects that were not captured in the interview and the impressions during data analysis were recorded in field notes and combined with the participants’ statements in the interview transcripts [32].

### 2.3. Data Analysis

**Figure 1:**
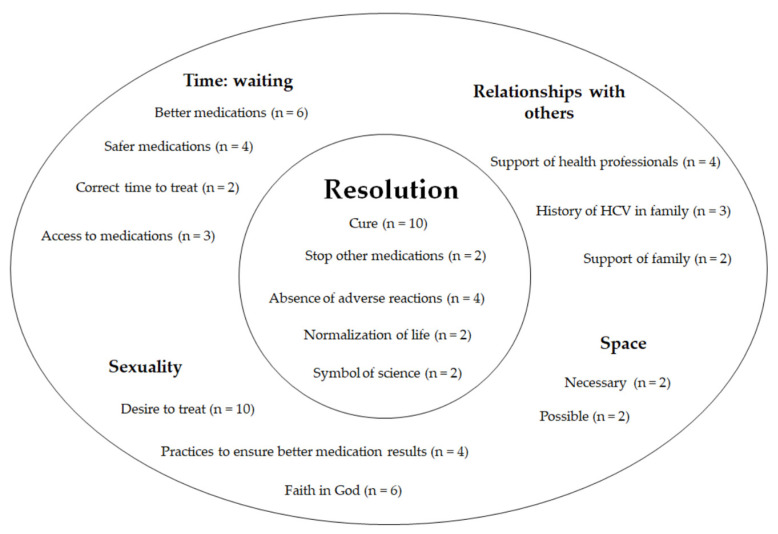
Ways of experiencing the use of DAAs and the essential structures of the experience: themes of the analysis and number of participants. (n = number of participants).

This study followed the theoretical framework systematized by Nascimento et al. [30,31]. From the definition of the objectives all the way to data analysis, phenomenology was used as the epistemological basis, phenomenology of existence as the paradigm and corporeality as the theory. According to these authors, patients with hepatitis C using multiple medications for different diseases might experience their medications as a sign of resolution, adversity, ambiguity and irrelevance (see Figure 1). Data analysis was carried out according to the following steps: detailed description of the phenomenon; phenomenological reduction; and reaching an understanding of the phenomenon [33,36].

The descriptive process began with the transcription of the interviews and multiple readings, followed by attentive listening to the recordings and review of the field data [36]. The next step was thematic analysis, based on a holistic approach, in which each data set was read and reread attentively. Next, with the help of NVivo Software v. 11 (QSR International, Burlington, VT, USA), a selective approach was utilized to search for essential phrases or statements to describe the phenomenon. After the units of meaning were identified, they were organized into themes and subthemes [33,36].

Merleau-Ponty did not propose a method for phenomenological research [37]; it is essential to develop a strategy that can capture this experiential dimension [38]. The use of structures of existence—time, space, relationships with others and sexuality—is one way to apply the theories of Merleau-Ponty in the context of health research [28,39] and to understand the daily use of medications [30]. In this regard, experiences with the use of second-generation antivirals, which were revealed through thematic analysis, were reorganized to encompass the essential structures of experience (Figure 1).

In this phase, phenomenological reduction was carried out. This method makes clear and explicit the theoretical, cultural and professional assumptions that influence the understanding of the lived phenomenon [40]. Furthermore, imaginative variation was employed, which is a type of mental experimentation wherein different aspects of experience are intentionally altered, excluding or including aspects of the phenomenon, until one reaches that which cannot be suppressed without destroying the phenomenon itself [36].

## 3. Results

The patients included in this study presented different backgrounds in terms of chronic HCV treatment. Learning about these stories increased our understanding of their experience with new second-generation DAAs. In this study, six were experienced patients, i.e., they had a history of at least one antiviral treatment, but they had not reached sustained virological response (SVR) or had relapsed; four had not received antiviral treatment because of contraindications for the schemes previously available and had been recently diagnosed and were also first-timers, but had no contraindications for treatment. Five patients presented cirrhosis as a complication of HCV infection and two (Zoé and Flora) had their own experiences with hepatitis C, but witnessed these experiences with a loved one who developed complications of HCV-associated liver disease (Table 1).

### 3.1. The Use of DAAs by Patients with Chronic Hepatitis C: The Experience of Resolution

The diagnosis of HCV infection directs the gaze to the physical body, and this disturbs patients’ relationships with the world and how they live their experiences, affecting their proper or phenomenal body. The fear of transmitting HCV to their partners and family members, and the anxiety that ensues from uncertainty about the evolution of the disease, are part of these patients’ experiences, as stated by one patient:

*“Worried about passing it on to others. Afraid of cutting myself. I have to think of everything, right. I’m worried about contaminating others.”*

(Ilma)

For this reason, second-generation DAAs were experienced as a solution to a problem in the physical and/or phenomenal body caused by the disease [30]:

*“Actually, the hepatitis medication is everything to me. Because it’s going to heal me.”*

(Rui)

Moreover, eliminating the virus was associated with normalizing all the changes he considered to be related to hepatitis:

*“The cure means not having the bug anymore. The virus. Not having it pestering me anymore.”*

(Rui)

This included the possibility of discontinuing the other medications he associated with his current clinical condition:

*“Because the hepatitis medication will stop as soon as the hepatitis is cured. I’ll stop taking the ascites ones. Because if I don’t have it, right… Automatically maybe even the glucose medication. Because my glucose is altered because of hepatitis… So that’s it, I’m waiting, and the cure will be everything to me.”*

(Rui)

In addition to normalizing the changes in the physical body, the expectation was that the cure caused by the medication will enable them to live normal lives [30], which created the possibility of moving on with life:

*“Then I’ll take care of my life, my family, my business. And not be so dependent.”*

(Rui)

However, in addition to resolving the problem for which it was indicated, it must not cause new problems, such as adverse reactions, and second-generation DAAs were considered very safe medications. The perception of the safety of the new medication was based on previous experiences with treatment, as stated by these patients:

*“Girl, that last one … I almost died with the last one. They made me very agitated, and all those blisters started popping up. I was in a real state!”*

(Ilma)

*“I’m not feeling a thing. This one is the same thing as the other one I was already on [for other diseases], I felt no difference at all.* *”*

(Flora)

Because it is an expected and desired treatment, the participants viewed the use of antivirals as beneficial, even if it can generate negative consequences, as reported by a patient who presented cirrhosis as a complication of HCV infection:

*“I’m still holding on well. My platelets dropped at first, but now they’ve gone up a bit. Because platelets are supposed to go down, right?”*

(Laís)

Although the medication is a symbol of the chemical, of what can somehow attack the naturally constituted body, Rui solved this impasse by avoiding knowledge about the risks associated with antivirals:

*“I read the leaflet. I know it doesn’t harm the heart. But it also doesn’t… So, I don’t know, but I think it has some side effects, but… I don’t think it’s in my best interest to know because I only want to get better. I just want to think about how great it is!”*

(Rui)

Last, the experience of the resolution of these medications was guided by science, based on the understanding that their availability is a technological advance:

*“I think about how science has advanced. That it has made such a blessed medication. And it keeps on trying and trying until it finds one that works!”*

(Ilma)

### 3.2. The Experience of Time: The Wait

Time for patients with chronic hepatitis C is marked by waiting, the stagnation of phenomenological time. Participants described waiting for: (1) better medications when they were not able to reach SVR; (2) safer medications, with fewer contraindications than those presented by previous treatment; (3) access to medications; (4) the most adequate time to use the medications; and (5) the cure and for their lives to go back to normal.

The patients experienced waiting for the development of new, more potent medications capable of eliminating the infection that had not been cured by previous treatment.

*“I did six months of treatment and felt very sick… My viral load went to zero. But less than two years later, the virus had relapsed. The doctor already told me about this medication that was going to arrive. She kept monitoring me, to see if we could get to where we are now. But there were times I was afraid. I was worried. But it worked out, thank God! And now I’m on this medication.”*

(Flora)

The participants reported the experience of waiting for safer medications that could be used. For some patients, the clinical context, namely, the complications of the disease, such as cirrhosis and its various clinical manifestations, imposes restrictions on the use of alternatives in previous treatments:

*“* *I couldn’t take the first one, because my platelets were so low, and I could die. There were too many side effects!”*

(Rui)

For others, the presence of numerous comorbidities also prevented the use of previous treatments:

*“The doctor said: The medication is here, but now that you’re on this other treatment, you can’t take it.”*

(Clara)

When science finally released the much-awaited effective and safe option, there were limitations in access to the treatment imposed by the high cost of medications, as described by Laís:

*“I didn’t have money to file a lawsuit, so I had to wait. Then the drugs arrived, but the Health Surveillance Agency blocked them. For a year… Then they released them again. But the government blocked them. Another year…”*

In the Brazilian public healthcare system, the high cost of treatment means that priority is given to patients who are in more advanced stages of liver disease and an experienced patient considered himself privileged and honored by receiving the treatment:

*“This is a gift. You understand? Because if it was available to everyone, cool. But it’s not! You know it’s not!”*

(João)

Still, frequent monitoring, which is necessary to ensure the effectiveness and safety of treatment, requires that patients reorganize their lives. In this regard, they must wait for the most appropriate time to begin, as reported by Nice:

*“The doctor talked about follow-up. I didn’t start treatment right then because my mother still was completely dependent on me. My mother passed away a year and a half later. But when I got here, the team thought my platelets were too low. So, the process took two years, waiting for this new medication.”*

Additionally, when treatment begins, a new wait begins—the wait for the cure:

*“It’s three months [treatment]. Twelve weeks. I’m in the fifth week, so halfway there, right?”*

(Laís)

The patient describes the treatment as a path to be traveled, demonstrating the interconnection between the dimensions of time and space in the subjective experience of using second-generation DAAs.

It is striking how patients experience the present as stagnated, a freezing of time because of all the waiting, summarized as follows:

*“When treatment stopped, I had to stop too, but always going after it.”*

(Ilma)

When the viral load is negative, i.e., when the patient is cured of chronic hepatitis C, the future flows to the present, and some patients are faced with the reality of the up to then forgotten cirrhosis:

*“I want to know what they’re going to do, because as soon as the virus is killed…then they’ll have to treat liver cirrhosis, right? The only thing on my mind before was treating the…virus. I was under the impression that when the virus was killed, everything would be over, the liver would regenerate. Then on second thought…cirrhosis cannot be regenerated… So, it’ll take even more time. They will have to wait, see if the liver reacts and if they will have to do something.”*

(Laís)

### 3.3. The Experience of Space—Possible Spaces and Necessary Spaces

For patients undergoing treatment, the experience of space can be described in two ways: there are possible spaces and necessary spaces [30]. The treatment and eradication of the virus means expanding possible spaces, allowing patients to resume their lives, as stated by one patient:

*“Then I will take care of my life, my family, my business.”*

(Rui)

For another, this means the security of not having to interrupt work and her source of income:

*“I’m afraid of doing nothing, of not being able to work. Being stuck in the house, it’s really bad.”*

(Ilma)

However, there were also necessary spaces, and in this regard, both the disease and the treatment are coercive. Follow-up care is intensified to ensure treatment outcomes, with weekly appointments at the outpatient unit at the beginning of treatment. This can be perceived as curtailed freedom for some patients, who need to reconcile treatment with their lives.

*“This routine is stressful, you don’t have time to take care of yourself (…). Because I have to get here at five in the morning! And I hate it. I wasn’t used to going to the doctor like this. For example, when it’s my day to come here, I am anxious the entire week! Just knowing that I will be stuck here, and I lose a whole day!”*

(Laís)

As Rui summed up:

*“Because you used to have a healthy life. But now you have a controlled life. By you, the doctors, by the situation.”*

### 3.4. Relationships with Others—Partnerships along the Way

In the context of treating HCV, trust in health professionals contributes to the experience of resolution revealed in this study. Ilma pointed to the interrelationships between space, time and others, when she very poetically defined the partnership of healthcare professionals who walked with her down the treatment path:

*“You never discouraged us; you always said a better medication would come along! Because I used to think that there was no solution and that you would discharge me, not care at all. But God blessed me, and you guys kept going. You walked beside us, right? Walking…”*

(Ilma)

Another aspect of relationships with others that arises in the context of treatment is experiences shared with both acquaintances and family members. For Zoé and Flora, witnessing a loved one experiencing the consequences of HCV infection generated in them the desire to undergo treatment.

*“He got cancer and it was all so fast. He was the kind of person who loved life, so I thought, man, he didn’t get the opportunity I’m being given now. I believe that if he had had the time, he would have gone after it! So, if I have this opportunity, I’d better grab it (crying)”*

(Flora)

### 3.5. Sexuality—“This Is a Medication I Want”

Regarding the pharmacological treatment of chronic hepatitis C, the patients reported a different involvement than the one that we, as healthcare professionals, witness when treating chronic diseases, as stated by one patient:

*“This is a medication I want. I want it, because God forbid, we should get to the point where I’ve seen others in, which is very difficult.”*

(Zoé)

The patients even reported changing the practices related to the use of medications in order to ensure the results of this treatment and clearly defines their effort to comply with the treatment:

*“I wasn’t usually strict with times, but now I think I’m better. Now I’ve created a routine, the alarm clock on my phone goes off and I’ll take the medication right away. And it’s not getting in my way.”*

(Flora)

*“It’s very hard for me to take medication. I’m taking these now without fail, asking God not to fail, dying to get rid of this…”*

(Zoé)

The motivation is living or making sure they are doing everything not to get in the way of eradicating the virus:

*“I’m the type of person who really loves living. I love life very much, I always joke and say, watch out, I’m going to live to be two hundred! And with this will to live, I have been dealing with this virus for twenty years now.”*

(João)

For Nice, this means making sure she is doing everything not to get in the way of eradicating the virus:

*“I don’t want to feel guilty if at the end they say, “It didn’t go down at all.” I will be aware that I did everything they prescribed, the way they told me to.”*

(Nice)

For some patients the motivation to abide by the treatment was their faith in God, that can be mixed with faith in the professionals:

*“I saw God’ hands in this on that first day. I said, He will take me to the end… And I worried no more! Positive thinking is very good for you, right?”*

(Laís)

*“The medication is the complement of what God already gave us… I think God has already cured me. You are godsent. The doctor of doctors is God.”*

(Rui)

## 4. Discussion

The present study demonstrated that for chronically HCV-infected patients utilizing the second-generation DAAs; this experience represented the resolution of the virus-related whole-body issues, i.e., a solution to a disturbance in the phenomenal body [29,30]. Several studies have indicated that the presence of the virus directs one’s gaze to the physical body, as a contaminated body [16,17,18,19]. This disturbs some patients’ relationships with the world and how they live their experiences, affecting the phenomenal body. The experience of patients with chronic hepatitis C is shaped by a number of factors: fear of transmitting the virus; difficult family relationships; stigma; anxiety due to uncertainty about the evolution of the disease; and physical, emotional or financial dependence [17,18,19,21,26,41,42]. Patients also reported the desire to end the stigma and shame caused by the infection and the fear of death [16,18,22,23,24]. The experience of witnessing friends or family members dying from cirrhosis or hepatocellular carcinoma motivated them to undergo treatment against the infection [16,18,20,22,23]. Harris [21] interconnected narratives of hope and expectation, corroborating the perception that the normalization of the phenomenal body provided by treatment was a driving force for patients with chronic hepatitis C.

From the time the disease was discovered in the late 1980s, up to the time of this study, science has provided four different modes of treatment for hepatitis C [7,8,9,10,11,12,13,14,15]. These therapies have gradually increased in effectiveness over time. It is worth noting that before the development of second-generation DAAs, antiviral therapies had important limitations due to numerous contraindications, such as uncontrolled depression, psychosis, epilepsy, retinal disease, autoimmune thyroid disease, decompensated liver disease and pregnancy (with PegIFN-a + ribavirin) [12]. However, DAAs have revolutionized the treatment of HCV infections, providing prospects for the comprehensive cure of chronic viral infections [43]. We believe that the peculiar historical development of hepatitis C treatment, combined with the personal experiences described by the participants in the conducted interviews, contributed to the experience of resolution found in this study.

The experience of time, marked by waiting, was central to this study. In a study by Harris [21], the period between diagnosis and the beginning of treatment was defined by one of the participants as “stasis” in which life came to a complete standstill. Sublette et al. [23] also described the strong desire of patients to receive treatment; however, contraindications for previous antiviral treatments triggered frustration in patients while they waited for therapy. In cases where there was no SVR, the certainty of living with the virus was defined by patients as a shadow, permeated by the future hope of a treatment capable of producing a cure [17].

The desire to use the medication does not arise just from the possibility of cure and normalizing the proper body. This innovative therapy has a high cost and represents an obstacle for many health systems around the world [21,26], and is not readily available. One of the strategies to determine priority in treatment of individuals with chronic hepatitis C has been based on the presence of more advanced liver disease and higher risk of complications [13,14,15,21,26,44]. Therefore, to be eligible for this treatment means to be in more advanced stages of liver disease, creating a situation referred by Whiteley et al. [26] (p. 5) as a “double-edged sword”.

Attentive waiting, i.e., periodical medical follow-up on those who do not meet the eligibility criteria for immediate pharmacological treatment significantly impacts the patients’ quality of life [17]. Some of these individuals had the virus and even though they did not present the feared complications, i.e., liver cirrhosis and hepatocellular carcinoma, they were at great risk. They were aware of this risk and how to decrease it; however, the solution was not available to all. Because of this awareness that they belonged to a select group to which therapy is not denied, patients expressed their gratitude, and considered themselves privileged and honored by receiving the treatment.

Together, these issues reinforce the need for an ethical discussion regarding treatment for chronic hepatitis C. From an economic point of view, treating all patients is extremely expensive. However, it is necessary to consider, on the one hand, the financial costs associated with treatment, and on the other, the costs involved in the treatment of complications of advanced liver disease, which also include liver transplants [45]. Beyond the economic benefits, Harris [21] described immeasurable benefits, such as being able to see and live with one’s children, being able to resume and maintain a love and sexual life and enjoying uninhibited play time with children and grandchildren. In this regard, Harris raises two important questions: “if treatment benefit can be purely conceptualized in clinical terms, and—in relation to treatment prioritization policies—is it ethical to do so?” [21] (p. 167). In the wake of this discussion, it is worth emphasizing that in Brazil, via the Unified Health System, this new technology has been incorporated, and therefore, access for all patients with chronic hepatitis C has increased.

The sick body, which needs medication to be freed of its condition, is hostage to the institutional space. This environment can be perceived in different ways, as welcoming or oppressive, but regardless, patients are confined to this space. This belonging to healthcare environments, which is necessary for conducting treatment, was a difficult aspect because of the need to reconcile care with professional engagements [23].

Furthermore, all the patients reported an absence of significant adverse reactions, in consonance with the findings of Whiteley et al. [25]. These experiences may have contributed to the perception of resolution found in this study, since this presupposes that physical and emotional body-related issues were solved, without the emergence of new problems, such as adverse reactions [30].

Some authors have described how patients face the challenges of treatment by developing coping strategies [18,23], characterizing the energy that drives them, as that which launches us into life situations, and acquire structures of conduct, which Merleau-Ponty called sexuality [31,37]. They sought to reframe the negative issues, using as a parameter the positive aspects associated with antiviral therapy, adopting routines to ensure the therapeutic effects [22,24], and using the faith in God and in the healthcare professionals as their support structure [18]. In this context, several patients completely reassessed their lives and chose their priorities [25], and faced with these challenges, established a treatment mentality, seeking to eradicate the virus and go back to a normal life.

Finally, another aspect that emerged in this study was the perception of chronic hepatitis C as an acute disease that can be cured. This perception contributed to revealing the experience of resolution, as a way of experiencing the use of DAAs, in juxtaposition with other ways of experiencing the use of chronic medications, such as adversity, ambiguity and irrelevance, which were observed with the use of other medications by these same patients [30].

If on the one hand this perception led to a different type of engagement, in which patients used all their resources to ensure the treatment’s success, on the other, it is worth emphasizing that this is a chronic disease, and several patients presented severe complications, which in themselves required specific and prolonged treatment. Paterson et al. [46] asserted that the healthcare provided to patients with chronic hepatitis C is often based on an acute care model, with a virus-centered reductionist approach, and that it recognizes its chronicity only in terms of the virus’s persistence. They also state that the way that care is provided to people with the disease influences how they experience the disease [21].

On the other hand, some authors argue that patients’ beliefs in this new era of treatment have not been strong enough to “challenge the well-founded cultural memory of HCV as a chronic condition” [26] (p. 9), and that although biological markers may indicate the absence of the virus, mental and physical scars can persist [21], given that people with hepatitis C see their disease in the context of their daily experiences and life objectives [46]. These results were not corroborated by the present study, although it was not longitudinal, and therefore, could not capture this dimension of the experience.

It should be noted that a possible limitation of the study is the setting in which the interviews were conducted, i.e., in an outpatient clinic visited by the patients. In this environment, participants may have experienced discomfort, which could have resulted in limited and incomplete answers.

## 5. Conclusions

The presence of chronic hepatitis C alters both the physical and phenomenal body and, in this context, the patients in this study experienced the use of second-generation antivirals to treat hepatitis C as providing resolution. This experience also permeated the following: (1) the experience of time—stagnant time, waiting for medication and the cure, symbolized by the medication; (2) the experience of spaces, understood as necessary and imposed spaces, which change their lives; (3) the experience of relationships with others, personified in this study by the support provided by healthcare professionals, but also as an incentive for treatment; (4) the experience of sexuality, when patients developed several coping strategies to deal with the challenges imposed by the treatment. These dimensions allow us to identify a convergence that clarifies and justifies the use of the medication as prescribed and indicated.

Finally, we emphasize the need to reassess the healthcare model for patients with hepatitis C so that it incorporates the dimensions of the experience of chronic illness, in which the eradication of the virus is only one of the steps in these patients’ treatment. The treatment of chronic HCV presents multiple challenges and increasing knowledge about the experiences of patients can contribute to a more holistic and empathetic approach by healthcare teams.

## Figures and Tables

**Table 1:** Participant’s clinic information.

Patient	Year of Diagnosis	Previous Treatment	HCV Medication History	HCV Complications
Rui	2011	No	Contraindication to previously available treatments	Yes
Nice	2011	No	Contraindication to previously available treatments	Yes
Laís	2001	Yes	Failure with two previous treatments	Yes
João	1994	Yes	Failure with one previous treatment	No
Zoé	2014	No	Recently diagnosed	Yes
Ilma	2006	Yes	Failure with two previous treatments	Yes
Flora	1997	Yes	Failure with one previous treatment	No
Bruna	2000	Yes	Failure with one previous treatment	No
Sara	2012	No	Recently diagnosed	No
Clara	2011	Yes	Failure with one previous treatment	No

## Data Availability

Not applicable.
